# Study of wheat (Triticum aestivum L.) breeding material potential for in vitro androgenesis

**DOI:** 10.18699/VJGB-23-117

**Published:** 2023-12

**Authors:** N.V. Petrash, T.N. Kapko, V.V. Sovetov

**Affiliations:** Siberian Research Institute of Plant Production and Breeding – Branch of the Institute of Cytology and Genetics of the Siberian Branch of the Russian Academy of Sciences, Novosibirsk, Russia; Siberian Research Institute of Plant Production and Breeding – Branch of the Institute of Cytology and Genetics of the Siberian Branch of the Russian Academy of Sciences, Novosibirsk, Russia; Siberian Research Institute of Plant Production and Breeding – Branch of the Institute of Cytology and Genetics of the Siberian Branch of the Russian Academy of Sciences, Novosibirsk, Russia

**Keywords:** doubled haploids, in vitro androgenesis, anther culture, Triticum aestivum L., heterosis, удвоенные гаплоиды, андрогенез in vitro, культура пыльников, Triticum aestivum L., гетерозисный эффект

## Abstract

Doubled haploid technology is a valuable biotechnological approach in plant breeding that enables one to quickly create new varieties through the single-stage production of homozygous lines. The aim of this study was to assess the indicators of in vitro androgenesis in the anther culture of the initial breeding material of varieties and combinations of F1 and F2 and to identify promising accessions with good responsiveness. For that purpose, the plant material that proved promising for the breeding programs of Siberian Research Institute of Plant Production and Breeding (SibRIPP<B) was used. Ten cultivars of common wheat and the F1 and F2 hybrids of nine combinations were evaluated for the main parameters of in vitro androgenesis such as the number of new formations, albino, green and all regenerated plants. Induction of androgenesis in vitro was carried out in anther culture in growth medium Chu (N6) containing 1 mg/l of growth regulator 2,4-D. The studied samples showed different responses to induction. The maximum level of new formations was found in F2 hybrids Novosibirskaya 15 × Lutescens ShT-335. The largest number of green plants was found in F1 Novosibirskaya 15 × Lutescens ShT-335. According to the results of variance analysis, a significant ( p < 0.01) influence of genotype on the studied traits was established. Varieties with good responsiveness to anther culture (Novosibirskaya 15) and lack of responsiveness to in vitro androgenesis (Novosibirskaya 31) were identified. Novosibirskaya 16 was characterized by a low regeneration capacity of new formations. A significant heterotic effect was revealed considering the number of new formations per 100 anthers among the hybrids of such combinations as Novosibirskaya 15 × Lutescens ShT-335, Novosibirskaya 15 × Lutescens 111/09, and Zagora Novosibirskaya × Obskaya 2. Novosibirskaya 15 was recommended for inclusion in crossings as a parental form that provides high hybrid responsiveness during in vitro androgenesis. The use of doubled haploid technology made it possible to quickly create DH-lines based on the breeding material.

## Abstract (ru)

Создание удвоенных гаплоидов – ценный биотехнологический подход в селекции растений, по- зволяющий ускоренно создавать новые сорта за счет одноэтапного получения гомозиготных линий. Целью настоящего исследования было проведение оценки показателей андрогенеза in vitro в культуре пыльников исходного селекционного материала сортов и комбинаций F1 и F2 и выявление перспективных образцов с хо- рошей отзывчивостью. В работе использован растительный материал, перспективный для селекционных про- грамм Сибирского научно-исследовательского института растениеводства и селекции – филиала ИЦиГ СО РАН. Десять сортов мягкой пшеницы и гибриды F1 и F2 девяти комбинаций скрещивания оценивали по основным параметрам андрогенеза in vitro: числу новообразований, числу альбиносов и зеленых растений-регенерантов и всех регенерировавших растений. Индукцию андрогенеза in vitro проводили в культуре пыльников на пита- тельной среде Chu (N6), в качестве регулятора роста использовали 1 мг/л 2.4-Д. У изучаемых образцов обна- ружен различный ответ на индукцию андрогенеза in vitro. Отмечен максимальный выход новообразований у гибридов F2 Новосибирская 15 × Лютесценс ШТ-335. Наибольшее количество зеленых растений-регенерантов обнаружено у F1 Новосибирская 15 × Лютесценс ШТ-335. По результатам дисперсионного анализа установлено достоверное ( p < 0.01) влияние генотипа на изучаемые признаки. Выявлены сорта с хорошей отзывчивостью в культуре пыльников (Новосибирская 15) и с отсутствием отзывчивости к андрогенезу in vitro (Новосибир- ская 31). Сорт Новосибирская 16 характеризовался низкой регенерационной способностью новообразова- ний. Среди гибридов значительный гетерозисный эффект отмечен по признаку «число новообразований на 100 пыльников» в комбинациях Новосибирская 15 × Лютесценс ШТ-335, Новосибирская 15 × Лютесценс 111/09, Загора Новосибирская × Обская 2. Сорт Новосибирская 15 рекомендован к включению в скрещивания как сорт, обеспечивающий высокую отзывчивость в андрогенезе in vitro гибридов. Применение технологии удвоенных гаплоидов позволило быстро создать DH-линии на основе изучаемого материала.

## Introduction

Common wheat (Triticum aestivum L.) is a critical cereal crop and the main source of vegetable protein for humans. According to the Food and Agriculture Organization of the United Nations (FAO), over 760 million tons of wheat was annually produced around the world in 2019–2021, with Russia having harvested around 78.8 million tons1. As the world’s population grows, increasing cereal production becomes a necessity. According to projections, the world cereal production is expected to reach 840 million tons by 2030 thanks to, among other things, higher wheat yields2.


Crops and livestock products: https://www.fao.org/faostat/en/#data/QCL



OECD/FAO (2021), OECD-FAO Agricultural Outlook 2021–2030, OECD Publishing, Paris.: https://doi.org/10.1787/19428846-en


As for breeding efforts, their main goal of is to develop new varieties combining high productivity, environmental plasticity, and resistance to diseases and other environmental stresses. Reaching this goal requires the use of new breeding material and advanced biotechnological methods

In addition to conventional wheat breeding methods including hybridization and multistage selection followed by a series of self-pollinations to achieve homogeneity and persistence, various optimization approaches have been widely used in recent years, such as production of DH (doubled haploids) lines. The latter are completely homozygous lines obtained by doubling the number of chromosomes in haploid plants. Their use accelerates the breeding process and makes it less laborious, while also providing unique genetic material for mapping populations, phenotyping, and genotyping (Hao et al., 2013; Hale et al., 2022).

DH make it possible to obtain homozygous lines from hybrid material in one generation, while conventional methods take five-six self-pollination generations. This allows plant breeders to produce a new variety in five-seven years and respond quickly to the needs of the grain market.

In recent years, researchers have focused on improving DH production protocols, which has allowed DH technology to become a fast and accurate tool for achieving homozygosity of the original breeding material (Maluszynski et al., 2003; Wędzony et al., 2009; Seguí-Simarro et al., 2021b). The research received a boost with the discovery of Datura anther culture’s ability to form haploid embryos and seedlings (Guha, Maheshvari, 1964). At present, DH production protocols are available for almost 400 species (Seguí-Simarro et al., 2021a). According to some authors, over 300 varieties have been produced using DH technologies in 12 plant species around the world (Forster, Thomas, 2005).

Doubled haploids may be obtained in vivo and in vitro. The use of in vivo systems implies obtaining a haploid embryo by parthenogenesis, pseudogamy, distant hybridization with subsequent elimination of alien pollinator chromosomes or as a result of intraspecific crosses (pollination by pretreated pollen, crosses with haploid induction lines). Chromosome doubling is a required step in all these DH production techniques. In vitro methods are based on obtaining plants from gametophyte cells by gynogenesis (cultivation of ovaries and flowers on nutrient media) or androgenesis (cultivation of anthers and isolated microspores) (Forster, Thomas, 2005; Seguí-Simarro et al., 2021b).

The isolated microspore culture and anther culture are widely used for production of haploids and DH plants in wheat breeding programs (Dunwell, 2010; Lantos et al., 2013; Seguí- Simarro et al., 2021a). DH production by in vitro androgenesis in anther culture (AC) is a simple and efficient method of obtaining pure lines (Castillo et al., 2015; Urazaliyev, 2015; Lantos, Pauk, 2016; Kolesnikova et al., 2021). The process is based on changing microspore development program from gametophyte way (pollen grain formation) to sporophyte, and the obtained embryo-like structures (ELS) and calluses are then used to grow regenerated plants (Embryological Foundations..., 2005). These plants are of significant breeding value because they develop from cells following the meiotic division, and thus have unique gene combinations. Haploid cells on the nutrient medium may undergo genome doubling and produce spontaneous DH plants with 100 % homozygosity as a result. In homozygous organisms, the effect of recessive genes can be seen along with that of dominant genes, which significantly accelerates genotype selection (Kasha, Maluszynski, 2003).

The efficiency of androgenesis in AC is affected by many factors, including donor growth conditions, microspore development stage, pretreatment conditions, nutrient medium composition, but genotype is what affects it the most (Tuvesson et al., 2000; Lantos, Pauk, 2020; Seguí-Simarro et al., 2021b; Hale, 2022). The success in obtaining androgenic regenerant plants is limited due to albinism and significant genotypic dependency (Li et al., 2013; Zhao L. et al., 2015). Genotypedependent variation in responsiveness can be seen both at intraspecific and interspecific levels. For example, hexaploid winter wheats show better in vitro androgenic responsiveness than the spring ones (Sharma et al., 2005; Lazaridou et al., 2016). A wheat-rye 1RS.1BL translocation has a positive effect on plant regeneration in in vitro androgenesis (Agache et al., 1989; Pershina et al., 2013; Timonova et al., 2022).

Additive, dominant, and epistatic relationships between genes responsible for inheritance of androgenic traits in AC were observed (Chaudhary et al., 2003; Dagüstü, 2008; Grauda et al., 2016). At the same time, some authors showed that androgenic responsiveness in AC followed a simple inheritance scheme and was controlled by dominant genes (El-Hennawy et al., 2011). B.E.S. Abd El-Fatah et al. (2020) demonstrated that additive effects prevailed over dominance effects in terms of genetic control of in vitro androgenic traits.

A viable strategy of overcoming genotypic dependency is to use breeding material with high in vitro androgenic responsiveness, i. e. one of the parents in the cross should induce the development of green regenerants in hybrids (Tuvesson et al., 2003; Kondic-Špika et al., 2011; Lantos, Pauk, 2020). Thus, it seems reasonable to assess the initial breeding samples and use the ones with good in vitro androgenic responsiveness in crosses.

The goal of the present study was to assess in vitro androgenic indicators in the anther culture of the initial breeding material from spring varieties of common wheat and combinations of F1 and F2, as well as identify promising accessions with good responsiveness.

## Materials and methods

Spring common wheat samples showing promise under the breeding program of Siberian Research Institute of Plant Production and Breeding (SibRIPP<B) – Branch of ICG SB RAS were used as breeding material. Nine combinations of F1 and F2 and ten parent varieties were selected for the assessment of in vitro androgenic responsiveness (Table 1).

**Table 1.**
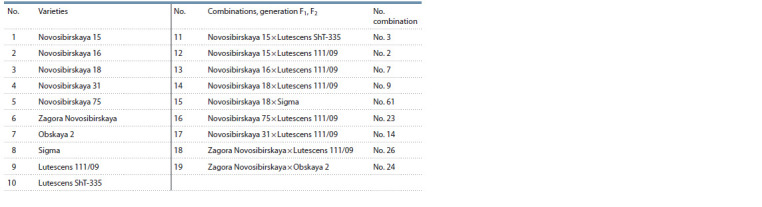
F1–F2 combinations and their parent varieties assessed with respect to in vitro androgenic responsiveness in the anther culture

Anther donor plants were grown on the field of Siberian Research Institute of Plant Production and Breeding in 2022. The spikes were harvested from leading shoots while most of the microspores were at the mid to late uninucleate stage. In terms of visual evidence, it meant that the middle of the spike was at the same height as the second top leaf sheath. Microspore development stage was identified using a Leica CME microscope (Leica Microsistems, Russia) in acetocarminestained cytological squash preparations.

The harvested spikes were stored in a temperature controlled container with cooling agents, transported to the laboratory, placed in test tubes with distilled water, and kept in a refrigerated thermostat TVL-K at +4 °С for seven days. After the cold pretreatment, the spikes were sterilized with wipes soaked in 70 % and then 96 % alcohol and transported to a biosafety box. The anthers were obtained from lateral flowers from the middle of the spike, with the average of about 50 anthers per spike. The experiments were performed in triplicate with one Petri dish for each measurement and with at least 100 anthers obtained for each accession.

The anthers from two spikes with the same genotype were inoculated in 100 mm Ø Petri dish with 15–20 ml of Chu’s N6 induction medium (Chu, 1978), 90 g/l sugars (sucrose: maltose in the ratio of 2:1); 100 mg/l myo-inositol; 1 mg/l 2,4-D, 0.5 mg/l kinetin, and 6 g/l plant agar. Petri dishes with anthers were incubated in the dark at 28 °С until the emergence of the first microspore-derived structures, and then at 25 °С for the further growth of the obtained structures. Following the incubation period of 30–40 days, the ELSs and calluses reaching 1.5–2 mm in diameter were placed in quantities of 3 to 5 in 28 mm Ø test tubes with Gamborg’s В5 medium (Gamborg et al., 1968), 30 g/l sucrose, 5 g/l plant agar without growth regulators. Plantlets regenerated under LED lights with photosynthetic photon flux density (PPFD) of 751.6 μmol/m2/s at 18–20 °С for 20–30 days with photoperiod of 16 hours.

Green plantlets with well-developed roots and leaves were taken out from the test tubes, with the remains of the nutrient medium thoroughly washed away from the roots, and planted into separate pots (0.8 l) with a mixture of coconut substrate, all-purpose soil, and vermiculite in the ratio of 3:1:1. The rooted plants were grown under the same LED lights at temperatures of 19–21 °С and humidity of about 50–60 %. The plants were grown to full maturity. Only the fertile plants (spontaneous DH) were selected for further study, while partially fertile or sterile plants were discarded.

The responsiveness of the AC was assessed using the following indicators: number of neoplasms (ELSs and calluses) per 100 isolated anthers (N/100A); number of albino plantlets per 100 isolated anthers (AP/100A); number of green plantlets per 100 isolated anthers (GP/100A); total plantlets per 100 neoplasms (TP/100N).

Statistical processing of the data was performed using Microsoft Exсel 2010. Analysis of variance was carried out using SNEDECOR software (Sorokin, 2004). True (Htr, %) and hypothetical (Hhyp, %) heterosis were calculated using Eqs. (1) and (2) based on (Omarov, 1975):

**Formula. 1.**
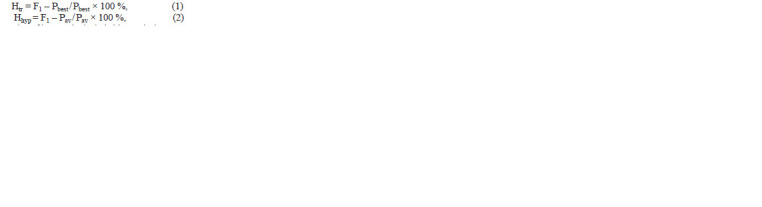
Formula. 1.

where F1 is the value of interest in the hybrid; Рbest is the same value in the best parent; Рav is the average value between parents (P1 + Р2)/2.

The degree of phenotypic dominance (Нр) acting as an inheritance indicator in the controlled crosses was calculated using Eq. (3) based on (Griffing, 1956):

**Formula. 2.**
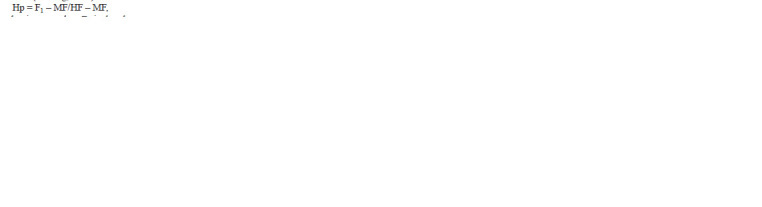
Formula. 2.

where Нр is the dominance value; F1 is the observed mean of F1; MF is the average attribute value between parents; and HF is the attribute value in the best parent. The interpretation was as follows: Hp > 1 was recognized as positive heterosis, Hp = 0.5–1.0 as positive dominance, Hp from +0.5 to –0.5 as intermediate inheritance, Hp = –0.5 to –1.0 as negative dominance, and Hp < –1.0 as negative heterosis. Inbreeding depression (ID %) was calculated using Eq. (4) based on (Pederson, 1971):

**Formula. 3.**
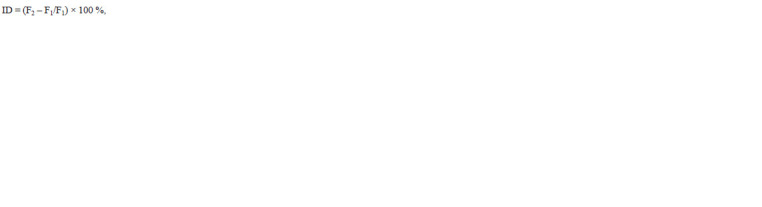
Formula. 3.

where ID is the inbreeding depression, F1 is the average attribute value in the first-generation hybrid family, F2 is the average attribute value in the second-generation hybrid family.

## Results and discussion

The success of DH technology in breeding programs depends on the genotype’s ability to regenerate green plants in in vitro androgenesis

In the present paper, the assessment of in vitro androgenic responsiveness is presented for 10 varieties and 9 combinations, generations F1 and F2. A total of 16,598 anthers have been isolated and placed in induction medium, with at least 100 anthers analyzed in triplicate for each accession. The single-factor analysis of variance showed a significant effect of genotype on all in vitro androgenic indicators of interest (Table 2).

**Table 2.**
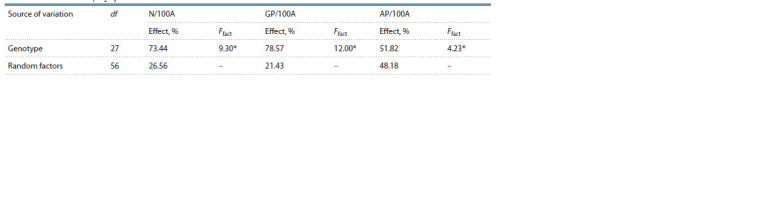
Single-factor analysis of variance for in vitro androgenic responsiveness indicators in the anther culture of wheat varieties and F1–F2 hybrids *p < 0.01 (Ftab. 0.99 = 2.18); df is the number of degrees of freedom; Ffact is the calculated Fisher test value; N/100A is the number of neoplasms per 100 anthers; GP/100A is the number of green plantlets per 100 anthers; AP/100A is the number of albino plantlets per 100 anthers

The studied samples showed a variety of in vitro androgenic responses (Table 3). The number of neoplasms per 100 isolated anthers (N/100A) indicates the quantity of structures (ELSs and calluses) developing from microspores. This attribute varied from 0 to 17.20 (Novosibirskaya 15 × Lutescens ShT- 335, F2), the average being 3.74. The average number of green regenerants per 100 anthers (GP/100A) was 1.45. Maximum values were observed in F1 Novosibirskaya 15 × Lutescens ShT-335 and Novosibirskaya 15 × Lutescens 111/09, at 12.15 and 12.50, respectively. The average number of albino plantlets per 100 anthers (AP/100A) was 0.63. Maximum values were observed in Novosibirskaya 15 (2.67), F2 Novosibirskaya 15 × Lutescens ShT-335 (2.40), and F1 Zagora Novosibirskaya × Obskaya 2 (2.92). The average total number of regenerants per 100 anthers was 2.08. Maximum values with prevalence of green regenerants were observed in F1 Novosibirskaya 15 × Lutescens ShT-335 and Novosibirskaya 15 × Lutescens 111/09. A total of 150 green plantlets were obtained in the experiment

**Table 3.**
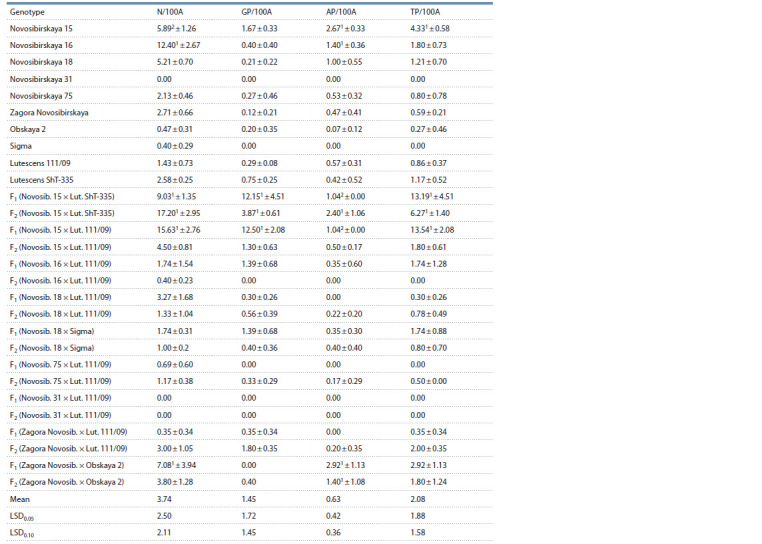
In vitro androgenic responsiveness indicators in the anther culture of wheat varieties and F1–F2 hybrids Notе. N – neoplasms; GP – green plantlets; AP – albino plantlets; A – anthers; TP – total plantlets; Novosib. – Novosibirskaya; Lut. – Lutescens. 1 Differences from the mean are significant at p = 0.05; 2 differences from the mean are significant at p = 0.10.

The analysis showed that high neoplasm production was not directly associated with a high number of regenerants. For instance, varieties Novosibirskaya 15 ( p <0.10) and Novosibirskaya 16 ( p< 0.05) both showed above average neoplasm production, but Novosibirskaya 15 also showed higher regeneration ability (TP/100A = 4.33, p < 0.05). Novosibirskaya 16 produced 12.40 neoplasms per 100 anthers with 1.80 regenerated plantlets per 100 anthers (see Table 3). This observation confirms the literature data that in vitro androgenic indicators are polygenically controlled and independently inherited (Ekiz, Konzak, 1994; Nielsen et al., 2015; Abd El-Fatah et al., 2020). Novosibirskaya 31 and, notably, its combinations in the first and second generations did not produce any structures, allowing us to assume that a non-responsive genotype worthy of further research has been discovered.

The ability of calli and embryo structures to regenerate into plantlets is reflected in the number of green regenerants per 100 neoplasms and the number of albino plantlets per 100 neoplasms (see the Figure). The experiment showed that the average number of regenerated green plantlets per 100 neoplasms was higher than the number of albino plantlets, the respective values being 26.41 and 18.74. The highest regeneration ability, with more than half of neoplasms regenerating into plants, was observed in hybrids F1 No. 3 (Novosibirskaya 15 × Lutescens ShT-335), No. 2 (Novosibirskaya 15 × Lutescens 111/09), No. 7 (Novosibirskaya 16 × Lutescens 111/09), No. 61 (Novosibirskaya 18 × Sigma), No. 26 (Zagora Novosibirskaya × Lutescens 111/09), and F2 No. 26 (Zagora Novosibirskaya × Lutescens 111/09) (see the Figure). Notably, the number of green regenerants per 100 neoplasms was above 100 for hybrid F1 No. 3 (Novosibirskaya 15 × Lutescens ShT-335), possibly due to secondary embryogenesis or an ELS developing into polyembryoids (structures with several shoot growth points (Seldimirova, 2009; Pershina et al., 2020)). Both mechanisms pro

**Fig. 1.**
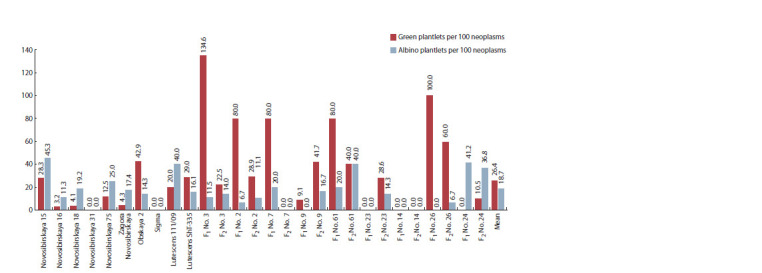
Percentage of green and albino plantlets per 100 neoplasms in in vitro androgenesis of wheat varieties and F1–F2 hybrids. No. 3 – (Novosibirskaya 15 × Lutescens ShT-335); No. 2 – (Novosibirskaya 15 × Lutescens 111/09); No. 7 – (Novosibirskaya 16 × Lutescens 111/09); No. 9 – (Novosibirskaya 18 × Lutescens 111/09); No. 61 – (Novosibirskaya 18 × Sigma); No. 23 – (Novosibirskaya 75 × Lutescens 111/09); No. 14 – (Novosibirskaya 31 × Lutescens 111/09), No. 26 – (Zagora Novosibirskaya × Lutescens 111/09); No. 24 – (Zagora Novosibirskaya × Obskaya 2); LSD0.05 (GP/100N) = 19.51; LSD0.05 (AP/100N) = 7.81.

duce clones or sister plants

Albinism acts as a limitation for DH production in in vitro androgenesis. Our experiment showed the prevalence of green plantlets in the total number of plantlets in varieties as follows: Obskaya 2, Lutescens ShT-335, F1 hybrids Novosibirskaya 15 × Lutescens ShT-335, Novosibirskaya 15 × Lutescens 111/09, Novosibirskaya 16 × Lutescens 111/09, Novosibirskaya 18 × Sigma, Zagora Novosibirskaya × Lutescens 111/09, and F2 hybrids Novosibirskaya 18 × Lutescens 111/09, Novosibirskaya 75 × Lutescens 111/09, Zagora Novosibirskaya × Lutescens 111/09 (see the Figure). It follows from the analysis of variance that around 50 % of albinism cases are genotype-related (see Table 2)

There are several factors increasing the chance of albinism, including genotype, donor growth conditions, cultivation conditions, medium composition, incompatibility of nuclear and plastid genomes, and plastid DNA deletions or mutations (Nielsen et al., 2015; Zhao P. et al., 2017). The high significance of the genotype’s effect on the number of albino plantlets is demonstrated in a number of papers (Lantos, Pauk, 2016; Castillo et al., 2019; Abd El-Fatah et al., 2020; Kanbar et al., 2020).

Genotypic dependency of albinism is associated with transcription activation of specific genes involved in chloroplast biogenesis at early stages (Mozgova et al., 2006; Canonge et al., 2021). Chloroplast DNA deletions were observed in albino plants, along with inhibited transcription of the nuclear genes coding for chloroplast-localized proteins, while levels of transcripts coding for proteins not present in chloroplasts were identical to those in green plants (Dunford, Walden, 1991).

To evaluate the prospects of using the studied varieties in further crosses, the heterosis effect in their hybrids was analyzed. Heterosis effect of in vitro androgenic responsiveness was described earlier, and its degree was shown to vary between genotypes (Ouyang et al., 1973; Ekiz, Konzak, 1994).

True (Htr) and hypothetical (Hhyp) heterosis, inheritance indicator (Нр), and inbreeding depression (ID %) were calculated based on the number of neoplasms per 100 anthers, since, according to the analysis of variance, genotype significantly contributes to this value (73.44 %, see Table 2) and directly affects the subsequent in vitro androgenic responsiveness indicators. Maximum hypothetical heterosis was observed in Zagora Novosibirskaya × Obskaya 2, and minimum, in Novosibirskaya 31 × Lutescens 111/09 (Table 4). True heterosis characterizes stronger manifestation of the trait in F1 compared to the best parent.

**Table 4.**
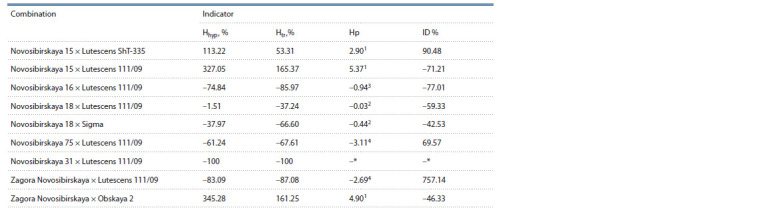
Heterosis effect and inheritance indicator for the number of neoplasms per 100 anthers in nine common wheat combinations Notе. Hhyp, % is the hypothetical heterosis; Htr,% is the true heterosis; ID % is the inbreeding depression; Hp is the degree of dominance; 1 positive heterosis; 2 intermediate inheritance; 3 negative dominance; 4 negative heterosis; * not available due to absence of neoplasms.

Maximum true heterosis of 100 % was observed in Novosibirskaya 15 × Lutescens 111/09, negative heterosis was observed in hybrids with Novosibirskaya 31 demonstrating androgenic non-responsiveness. Significant negative Htr was also observed in Novosibirskaya 16 × Lutescens 111/09 and Zagora Novosibirskaya × Lutescens 111/09.

Analysis of the inheritance indicator showed positive heterosis for Novosibirskaya 15 × Lutescens ShT-335, Novosibirskaya 15 × Lutescens 111/09, Zagora Novosibirskaya × Obskaya 2. Intermediate inheritance was observed in combinations with Novosibirskaya 18. Negative dominance was observed in Novosibirskaya 16 × Lutescens 111/09, and negative heterosis, in Novosibirskaya 75 × Lutescens 111/09 and Zagora Novosibirskaya × Lutescens 111/09.

The degree of manifestation of in vitro androgenic attributes varies between F1 and F2 hybrids. The first generation outperformed the second one in neoplasms per 100 anthers in Novosibirskaya 15 × Lutescens 111/09, Novosibirskaya 16 × Lutescens 111/09, Novosibirskaya 18 × Lutescens 111/09, Novosibirskaya 18 × Sigma, Zagora Novosibirskaya × Obskaya 2. Inbreeding depression was observed in Novosibirskaya 15 × Lutescens ShT-335, Novosibirskaya 75 × Lutescens 111/09, Zagora Novosibirskaya × Lutescens 111/09 (see Table 4). Negative ID % value shows that F1 hybrids outperform F2 in terms of manifestation of the attribute.

To summarize the analysis of the inherited ability to produce structures from microspores in various combinations, it is worth focusing on positive values observed for combinations with Novosibirskaya 15. These results agree with the previously obtained data on the responsiveness of F1 and F2 hybrids Obskaya 2 × Novosibirskaya 15 compared to parent varieties (Petrash et al., 2022). Studying the inheritance patterns in multiple combinations makes it possible to estimate positive in vitro androgenic responsiveness in hybrids to ensure effective pair selection for crosses under future breeding programs using doubled haploid technology

## Conclusion

The goal of the paper was to study the potential of the initial breeding material from the perspective of in vitro androgenesis in 10 different common wheat varieties and 9 combinations of F1 and F2, with a total of 28 genotypes analyzed. The androgenic indicators analyzed included the number of neoplasms (ELSs and calluses), green plantlets, albino plantlets, and the total number of regenerated plants.

As a result, the varieties showing in vitro androgenic responsiveness (Novosibirskaya 15) and non-responsiveness (Novosibirskaya 31) in the anther culture have been identified. Novosibirskaya 16 was characterized by low neoplasm regeneration ability. A significant heterosis effect was observed in hybrids Novosibirskaya 15 × Lutescens ShT-335, Novosibirskaya 15 × Lutescens 111/09, Zagora Novosibirskaya × Obskaya 2. Positive heterosis in terms of neoplasms per 100 anthers was observed in combinations with Novosibirskaya 15, and intermediate inheritance, in combinations with Novosibirskaya 18. Novosibirskaya 15 is recommended for inclusion into crosses as a variety ensuring high in vitro androgenic responsiveness in hybrids compared to the second parent. Doubled haploid technology made it possible to use the discussed hybrid material to produce DH lines, which are now being tested in the field.

## Conflict of interest

The authors declare no conflict of interest.
